# From Molecular Classification to Targeted Therapeutics: The Changing Face of Systemic Therapy in Metastatic Gastroesophageal Cancer

**DOI:** 10.1155/2015/896560

**Published:** 2015-02-17

**Authors:** Adrian Murphy, Ronan J. Kelly

**Affiliations:** ^1^Upper Aerodigestive Malignancies Division, Sidney Kimmel Comprehensive Cancer Center at Johns Hopkins, Baltimore, MD 21231, USA; ^2^Gastroesophageal Cancer Therapeutics Program, Sidney Kimmel Comprehensive Cancer Center at Johns Hopkins, Bunting Blaustein Cancer Research Building, 1650 Orleans Street, Room G93, Baltimore, MD 21231, USA

## Abstract

Histological classification of adenocarcinoma or squamous cell carcinoma for esophageal cancer or using the Lauren classification for intestinal and diffuse type gastric cancer has limited clinical utility in the management of advanced disease. Germline mutations in E-cadherin (*CDH1*) or mismatch repair genes (Lynch syndrome) were identified many years ago but given their rarity, the identification of these molecular alterations does not substantially impact treatment in the advanced setting. Recent molecular profiling studies of upper GI tumors have added to our knowledge of the underlying biology but have not led to an alternative classification system which can guide clinician's therapeutic decisions. Recently the Cancer Genome Atlas Research Network has proposed four subtypes of gastric cancer dividing tumors into those positive for Epstein-Barr virus, microsatellite unstable tumors, genomically stable tumors, and tumors with chromosomal instability. Unfortunately to date, many phase III clinical trials involving molecularly targeted agents have failed to meet their survival endpoints due to their use in unselected populations. Future clinical trials should utilize molecular profiling of individual tumors in order to determine the optimal use of targeted therapies in preselected patients.

## 1. Introduction

While the incidence of gastric cancer is decreasing in the United States, the rates of esophageal cancer are increasing. In 2014 it is anticipated that 22,220 and 18,170 patients will be newly diagnosed with gastric cancer and esophageal cancer, respectively, while 26,440 men and women will die as a result of upper gastrointestinal (GI) tumors [1]. Gastroesophageal cancer remains endemic in many parts of the world with an estimated new cancer incidence of 1,471,000 or 11.6% of the global cancer burden and a death rate annually of 1,144,000 people or 15.1% of cancer-related deaths worldwide. Taken together, esophageal cancer and gastric cancer are second only to lung cancer in incidence and in mortality [2]. Despite these figures, there has been a dearth of scientific breakthroughs in these tumor types which have resulted in significant survival advantages.

The location of upper GI tumors in western countries has changed dramatically in recent years. Distal gastric cancer, which previously predominated, has become uncommon, whereas the incidence of tumors of the gastric cardia and gastroesophageal junction has increased dramatically. Prior to the 1970s, distal esophageal adenocarcinomas were uncommon, representing 0.8–3.7% of esophageal cancers [3]. Over the past three decades, there has been a sevenfold increase in the incidence of esophageal adenocarcinoma among US white males, now accounting for more than half of the cases of esophageal cancer. Through the 1980s, the increases in the rates of these tumors have been on the order of 5–10% per year, a faster pace than for virtually any other cancer in the United States [3]. This has been attributed to declining chronic infection rates by* Helicobacter pylori* and an increased incidence of gastroesophageal reflux disease and obesity [4–6].

## 2. Cytotoxic Chemotherapy for Gastroesophageal Cancer

The treatment of metastatic gastroesophageal junction and gastric cancer has been poorly investigated and at present there is no single chemotherapeutic regimen that is considered standard first-line treatment. Various combinations of platinum-based doublets and triplets have been prescribed depending on patient performance status and physician preference. Current therapy for advanced disease is limited and survival rarely exceeds one year despite aggressive treatment with modern chemotherapy. Second-line regimens have, until recently, been considered ineffective. A number of different drugs (e.g., alkylating agents, platinum compounds, 5 FU, and taxanes) are available for the treatment of gastroesophageal cancer but no means of selecting therapy based upon the biology of the tumor is currently available (Table 1). HER2 status remains the only validated molecular marker which influences clinician decision-making in the metastatic setting. At present the combination of a platinum and fluorouracil, either alone or in combination with a third drug such as epirubicin or a taxane, constitutes the most effective treatment option in the first-line metastatic setting [7]. Standard first-line options include DCF (docetaxel, cisplatin, and 5 FU), ECF/EOX (epirubicin, cisplatin/oxaliplatin, and 5 FU/capecitabine), or FOLFOX (5 FU, oxaliplatin) [8–10]. Additional FDA approved 2nd line agents include docetaxel, paclitaxel, and irinotecan [11–14].

## 3. Molecular Classification of Gastric Cancer

Molecular profiling studies have been performed using gene expression or DNA sequencing and have identified distinctive molecular signatures which may predict responsiveness to systemic therapies. Earlier studies in gastric cancer concentrated on molecular signatures which characterized the processes of tumorigenesis. Microarray-based gene expression profiling identified characteristic expression patterns which readily discern premalignant from malignant tissues [15]. Chronic gastritis tissue expressed a marked mitochondrial gene expression signature possibly as a response to* H. pylori* infection, for example,* NDUF* (NADH dehydrogenase), whereas intestinal metaplastic tissue expressed a more transformed phenotype including many intestinal differentiation genes which were not expressed in tumor tissues, for example,* CDX1*,* MYO1A*, and* villin A*. Later studies examined whether the molecular signature could predict sensitivity to chemotherapy [16]. Genomic subtypes (intestinal and diffuse) identified from* in vitro* studies in gastric cancer and validated in primary tumors were found to be prognostic of survival and had the ability to predict sensitivity to 5 FU and/or platinum agents. It was possible to detect these subtypes by immunohistochemical analysis of* LGALS4* and* CDH17* expression. These studies may ultimately identify predictive biomarkers allowing physicians to personalize chemotherapy selection in gastric cancer.

Molecular profiling has been extended in an attempt to predict responsiveness to targeted therapies [17]. Gene expression patterns were analyzed with advanced bioinformatics tools to identify molecular signature subtypes which predicted response to inhibitors of the PI3K/Akt/mTOR pathway.

**Figure 1:**
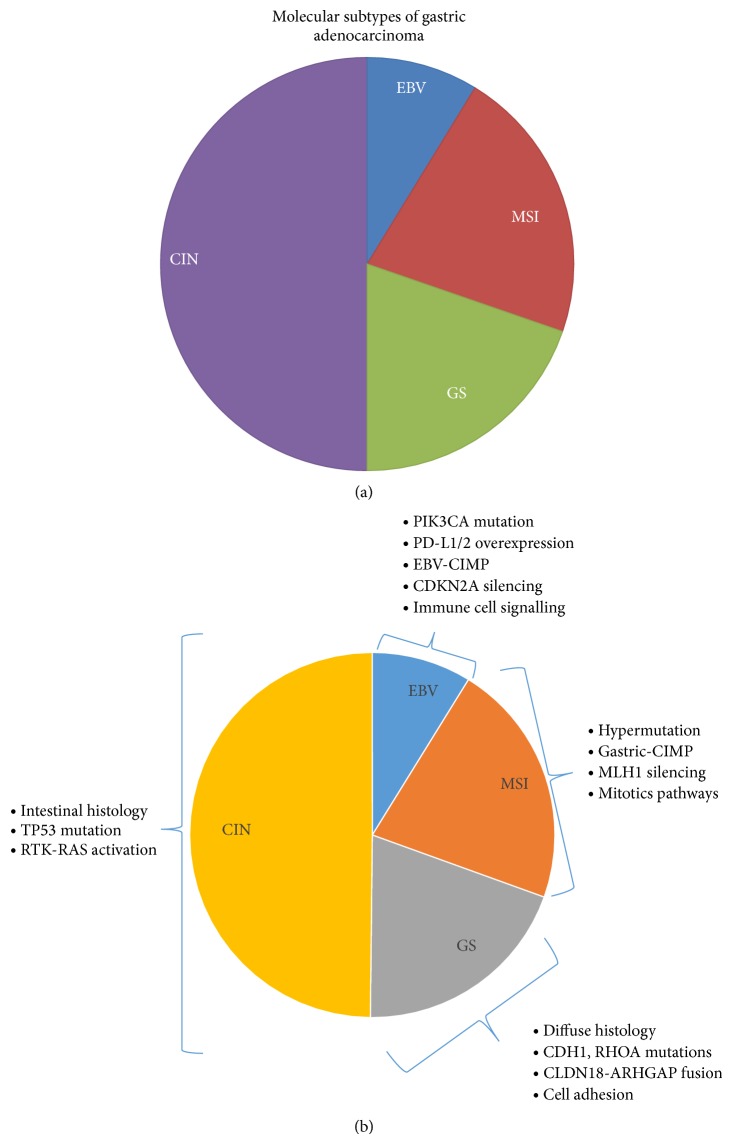
(a) Molecular classification of gastric adenocarcinomas. Primary gastric adenocarcinomas (*n* = 295) were analyzed in the TCGA project and found to have four main subtypes: CIN (chromosomal instability) 49.8%, GS (genomically stable) 19.6%, MSI (microsatellite instability) 21.7%, and EBV (Epstein-Barr virus), positive 8.8%. Adapted from data in TCGA [18]. (b) Characteristics of molecular subtypes of gastric cancer. Adapted from data in TCGA [18]. The key features of each molecular subtype are listed adjacent to the representation of subtype.

The Cancer Genome Atlas Research Network (TCGA) has recently performed a comprehensive molecular characterization of gastric tumors from 295 patients who had not been treated with prior chemotherapy or radiotherapy [18]. Detailed genetic analysis was performed using array-based somatic copy number analysis, whole-exome sequencing, array-based DNA methylation profiling, mRNA sequencing, microRNA sequencing, and reverse-phase protein arrays. It has proposed four subtypes (Figure 1(a)): (1) tumors positive for Epstein-Barr virus, (2) microsatellite unstable tumors, (3) genomically stable tumors, and (4) tumors with chromosomal instability.

EBV-associated tumors were shown to have a higher prevalence of DNA hypermethylation than any other tumor reported by the TCGA. All EBV-positive tumors displayed* CDKN2A* promoter hypermethylation and 80% had* PIK3CA* mutations. In addition, PD-L1/2 expression was elevated in EBV-positive tumors suggesting a role of targeted immunotherapy in this subset of gastric tumors. Microsatellite unstable (MSI) tumors generally lacked targetable amplifications although mutations in* PIK3CA*,* HER2*,* HER3*, and* EGFR* were noted.* BRAF* (V600E) mutations were not seen in gastric MSI tumors unlike its counterpart in colorectal cancer. Genomically stable gastric tumors are enriched for the diffuse histological variant and have newly described mutations in* RHOA* which acts through several effectors to control actin-myosin-dependent cell contractility and motility. In addition, a recurrent interchromosomal translocation (between* CLDN18* and* ARHGAP26*) implicated in cell motility was found in genomically stable gastric tumors. Almost half of gastric tumors demonstrated chromosomal instability which was characterized by marked aneuploidy and focal amplification of receptor tyrosine kinases such as amplification of* VEGFA* and frequent amplifications of cell cycle mediators (*CCNE1*,* CCND1*, and* CDK6*). This study has considerably added to our knowledge of the molecular subtyping of gastric cancer and hopefully will permit improved patient selection for clinical trials in the future.

## 4. Molecular Classification of Esophageal Cancer

Chromosomal aberrations leading to gene dysregulation have been reported in esophageal cancer including amplifications on 8q and 17q mapped to the* C-MYC* and* ERBB2* oncogenes [19, 20].

The role of* MYC* in the pathogenesis of esophageal cancer is not well defined and additional research is required. Loss of heterozygosity of TP53 occurs in greater than 50% of cases of esophageal cancer and is considered a strong predictor of disease progression [21–23]. In addition, two genes reported to have homozygous deletions in esophageal cancer are* p16/CDKN2A* and* FHIT* [24]. Abeloff et al. performed an integrative analysis of array-comparative genomic hybridization and matched gene expression profiling to reveal novel genes with prognostic significance in esophageal adenocarcinomas [25]. The authors identified 17 common regions (>5%) of gain and 11 common regions of losses in 56 resected specimens with associated long-term clinical follow-up data. Novel regions identified included loci 11p13 and 21q21.2.

Genes with high copy number and expression correlations included two deletions (*p16/CDKN2*,* MBNL1*) and four gains (*EGFR*,* WT1*,* NEIL2*, and* MTMR9*). These genes individually (*P* < 0.06) and collectively had prognostic significance (*P* = 0.008). A host of additional genes have been studied for mutations in esophageal cancer, but in most of these single gene studies, very few mutations have been identified. In an effort to perform a comprehensive evaluation of all coding regions for mutations, Agrawal et al. performed a comprehensive study of esophageal cancer exomes including both adenocarcinomas and squamous cell carcinomas [26]. Inactivating mutations of* NOTCH1* were identified in 21% of esophageal squamous cell carcinomas but not in adenocarcinomas.

Dulak et al. conducted an analysis of somatic copy-number alterations using high-density genomic profiling arrays in 296 esophageal and gastric cancers [27]. Amplified genes were noted in 37% of gastric/esophageal tumors, including* ERBB2*,* FGFR1*,* FGFR2*,* EGFR*, and* MET*, suggesting that some of these may be viable targets in esophageal cancer although amplification of some of these may be more prevalent in gastric tumors.

## 5. Molecular Targets in Advanced Disease

### 5.1. HER2

HER2 is a protooncogene which is encoded by* ERBB2* found on chromosome 17. It belongs to the HER family of membrane-bound receptor tyrosine kinases which is responsible for the initiation of cell signaling pathways via phosphoinositide 3-kinase, phospholipase C, and mitogen-activated protein kinase [28]. While originally known for its effects in breast cancer, HER2 overexpression has been shown to result in worse prognosis in gastric cancer [29, 30] although there are conflicting studies which suggest that it has no effect or is even beneficial in terms of prognosis [31, 32].

The TOGA trial was a phase III prospective trial which demonstrated the benefits of adding trastuzumab, a humanized monoclonal antibody targeting HER2, to a platinum-based doublet in the presence of HER2 IHC 2+ or FISH amplified metastatic gastroesophageal or gastric cancer [33]. In this trial, 594 patients were randomly assigned to study treatment (trastuzumab plus chemotherapy, *n* = 298; chemotherapy alone, *n* = 296), of whom 584 were included in the primary analysis (*n* = 294; *n* = 290). Median overall survival was 13.8 months (95% CI 12–16 months) in those assigned to trastuzumab plus chemotherapy compared with 11.1 months in those assigned to chemotherapy alone (hazard ratio 0.74; 95% CI 0.60–0.91; *P* = 0.0046). Unfortunately only 15–20% of patients with esophageal or gastroesophageal tumors are HER2 positive [32] and intestinal-type disease and tumors at the gastroesophageal junction and proximal stomach have higher HER2 expression [33]. The benefit of trastuzumab was confined to those with IHC 2+/3+ and FISH positivity.

The practice of maintenance trastuzumab and continuing trastuzumab until evidence of disease progression are commonplace in the management of breast cancer [34, 35] but there is a lack of data indicating that this is a successful strategy in gastroesophageal cancer although a Japanese trial is currently investigating this approach [36]. The development of resistance to trastuzumab has prompted investigating alternative drugs which target HER2. Lapatinib is an oral dual tyrosine kinase inhibitor which targets EGFR and HER2 domains resulting in blocked autophosphorylation and downstream signaling [37]. The phase III TYTAN study compared paclitaxel with or without lapatinib in HER2 positive gastric cancer in the second-line setting in Asian patients [38]. Median overall survival was 11 months with paclitaxel plus lapatinib compared to 8.9 months with paclitaxel alone (*P* = 0.1044). There was also no significant difference in PFS or TTP. Pertuzumab is a monoclonal antibody which binds to HER2 preventing its dimerization with other HER receptors [39]. It is currently the subject of a phase III trial (JACOB) comparing pertuzumab/trastuzumab + chemotherapy to pertuzumab + chemotherapy [40]. Trastuzumab emtansine (T-DM1) is an antibody-drug conjugate combining trastuzumab and DM1 which is a cytotoxic/microtubule polymerization agent [41]. T-DM1 binds to the HER2 receptor resulting in internalization of the DM1-receptor complex which results in the inhibition of cell division/growth and ultimately cell death [42]. The ongoing GATSBY trial is currently investigating T-DM1 versus a taxane in patients with previously treated HER2-positive metastatic or locally advanced gastric cancer [43].

### 5.2. EGFR

The epidermal growth factor receptor (EGFR) is known to play an important role in the initiation of signaling transduction cascades via phosphorylation of numerous cellular proteins [44]. A meta-analysis of 7 studies concluded that EGFR expression correlates with decreased survival in gastric cancer [45]. In addition, preclinical data suggest that a subset of gastric cancers (20%) with EGFR amplification and overexpression can respond to cetuximab therapy [46]. Cetuximab is a monoclonal antibody directed against the EGFR receptor and was shown to improve outcomes in* kras* wild-type metastatic colorectal cancer [47]. The EXPAND study was a phase III trial which studied capecitabine and cisplatin with or without cetuximab in the first line setting in advanced gastric cancer [48]. Median PFS for chemotherapy/cetuximab was 4.4 months versus 5.6 months for those who received chemotherapy alone (HR = 1.09, 95% CI 0.92–1.29, *P* = 0.32). Adding cetuximab to this chemotherapy combination also did not improve overall survival (9.4 months in both arms, HR = 1.0, 95% CI 0.87–1.17, *P* = 0.95). The phase III REAL-3 trial compared EOX (epirubicin, oxaliplatin, and capecitabine) with or without panitumumab, a fully human antibody targeting the EGFR receptor [49]. Median overall survival in the chemotherapy group was 11.3 months compared to chemotherapy + panitumumab which was 8.8 months (HR = 1.37, 95% CI 1.07–1.76, *P* = 0.013). An additional EGFR-targeting drug matuzumab also gave disappointing results in advanced esophagogastric cancer [50]. A combination of ECX chemotherapy/matuzumab failed to improve overall survival (9.4 months for matuzumab group compared with 12.2 months, HR = 1.02, 95% CI 0.61–1.70, *P* = 0.945).

While these results may highlight the lack of importance of the EGFR pathway in esophagogastric cancer, it is important to note that many negative studies were conducted in an unselected population which may explain their negative results.

### 5.3. C-MET

C-MET has been proposed as a promising new target in advanced disease and a number of phase III trials are now in progress combining MET inhibitors with chemotherapy in the first-line setting for metastatic gastroesophageal cancer. C-MET is a receptor tyrosine kinase which interacts with its ligand HGF (hepatocyte growth factor) [51]. It has been found to be dysregulated in gastric cancers and is involved with tumor proliferation, invasion, and angiogenesis and has antiapoptotic functions in cancer cells [52, 53]. Tumors which harbor high C-MET expression are more likely to have poor survival rates [54]. In a recently reported phase II study, the anti-HGF monoclonal antibody rilotumumab was combined with chemotherapy with progression-free survival (PFS) as the primary endpoint. PFS was 5.7 months in rilotumumab treatment arms versus 4.2 months in the placebo group (HR = 0.60, 80% CI 0.45–0.79; *P* = 0.016) [55]. Unfortunately a significant increase in overall survival was not reported although a phase III study is ongoing comparing rilotumumab alone or in combination with cisplatin/capecitabine in the first-line setting [56]. Tivantinib, although originally regarded as a c-MET inhibitor, has been shown to function independently of the c-MET pathway. Studies on lung cancer cell lines have shown that tivantinib does not inhibit cellular MET activity or downstream phosphorylation of Akt or ERK 1/2 in met-dependent cell lines [57]. Another preclinical study has shown that tivantinib inhibits microtubule polymerization independent of c-MET [58]. However, tivantinib has shown promising efficacy as a single agent in a phase II study meriting further study in combination with chemotherapy in a phase III design [59].

## 6. Antiangiogenic Therapy

Angiogenesis is largely recognized as an important aspect of tumorigenesis and preliminary clinical studies suggested a clinical benefit in the addition of bevacizumab, a monoclonal antibody against VEGF-A, in combination with chemotherapy in gastric cancer [60, 61]. The failure of bevacizumab to improve overall survival in the phase III AVAGAST trial was a disappointing development although interestingly it did appear from a subset analysis that a western population may derive some benefit [62, 63]. When subset analyses were performed in the AVAGAST trial, it appeared that those with type 3 (distal nondiffuse) gastric cancer and those from European/American populations derived more benefit from bevacizumab than other gastric cancer subtypes or patients from Asian/Pacific populations. The VEGFR-2 (vascular endothelial growth factor receptor-2) antagonist ramucirumab, as reported in the REGARD trial, demonstrated modest activity in patients with advanced gastric or gastroesophageal junction adenocarcinoma who had disease progression after first-line platinum-containing or fluoropyrimidine-containing chemotherapy [64]. Median overall survival was 5.2 months (IQR 2.3–9.9) in patients in the ramucirumab group and 3.8 months (IQR 1.7–7.1) in those in the placebo group (HR = 0.776, 95% CI 0.603–0.998; *P* = 0.047). The subsequently reported RAINBOW trial investigated paclitaxel ± ramucirumab in patients with metastatic GEJ or gastric adenocarcinoma who had disease progression on or within 4 months after first-line platinum- and fluoropyrimidine-based combination therapy [65]. The primary endpoint was overall survival (OS). Median OS was 9.63 months for ramucirumab + paclitaxel compared to 7.36 months for paclitaxel alone (HR = 0.807, 95% CI 0.678–0.962, *P* = 0.017). Based on these results the combination of ramucirumab + paclitaxel is expected to become a standard of care treatment regimen in the second-line setting for metastatic upper GI tumors. The success of ramucirumab in the 2nd line setting has prompted its clinical investigation in the first line setting. When ramucirumab was combined with FOLFOX in the first-line setting, it did not improve median progression-free survival (6.4 versus 6.7 months, HR = 0.98, 95% CI 0.69–1.37, *P* = 0.89) or overall survival (11.7 versus 11.5 months, HR = 1.08, 95% CI 0.73–1.58) in patients with advanced gastric/GE junction tumors [66]. Clinical trials investigating alternative combinations of chemotherapy with ramucirumab in the first-line setting are ongoing.

Prior to the success of ramucirumab, tyrosine kinase inhibitors had been explored in their roles as antiangiogenic agents in the treatment of esophagogastric tumors. Several tyrosine kinase inhibitors with known antiangiogenic activity are already in use for other tumor types, for example, renal cell cancer. Sunitinib was studied in a phase II trial in patients with advanced gastric or GE junction tumors who had progressed on chemotherapy [67]. The clinical benefit rate was 7.7% with 32.1% of patients exhibiting disease stability. By intent-to-treat analysis, median overall survival was 6.8 months (95% CI 4.4–9.7 months). The addition of docetaxel to sunitinib resulted in a higher objective response rate (41.1% versus 14.3%, *P* = 0.02) although this study did not meet its primary endpoint of prolonging time-to-progression [68]. Sorafenib, a multitarget TKI of BRAF, VEGF, and PDGFR, was combined with oxaliplatin in a phase II trial in patients who had progressed on first-line cisplatin/fluoropyrimidine chemotherapy [69]. This study did not meet its primary endpoint of efficacy and showed median PFS of 3 months (95% CI 2.3–4.1 months) and median overall survival of 6.5 months (95% CI 5.2–9.6 months).

## 7. Cancer Cell Stemness

The characteristic of cancer cells' ability to grow indefinitely has led to the theory that they share common underlying mechanisms with stem cells [70]. BB1608 is an orally available cancer cell stemness inhibitor whose exact target has not been elucidated [71]. It has been shown to inhibit the Stat3, *β*-catenin, and Nanog pathways. To date, a phase Ib study has been conducted combining BB1608 and paclitaxel in advanced cancers and of 5 patients enrolled with refractory gastric/GEJ tumors, 2 had partial responses, 1 had stable disease with 25% regression, and 2 had prolonged disease stability ≥24 weeks.

## 8. Hedgehog Inhibitors

The sonic hedgehog (SHH) pathway is crucial for normal cell differentiation and aberrant function affects gastric cell proliferation, migration, and invasion [72]. A phase II trial combined vismodegib (SHH pathway inhibitor) with FOLFOX in patients with advanced gastric and GE junction tumors [73]. Patients were treated in the first-line setting and the primary endpoint of median PFS was not met in the intention-to-treat population (11.5 months for FOLFOX/vismodegib versus 9.3 months for FOLFOX alone, 95% CI 8.5–14.4, *P* = 0.34). However, the expression of CD44, a gastric cancer stem cell marker, was associated with improved survival in the group who received the SHH inhibitor suggesting that SHH inhibition may only be effective in those with high CD44 expression [74].

## 9. IGFR Targeted Therapy

Insulin-like growth factors (IGF) are potent hormones which regulate cell proliferation, differentiation, and survival via endocrine, paracrine, and autocrine pathways [75]. Increased IGF-1R signaling, a ubiquitously expressed tyrosine kinase receptor, is associated with downstream activation of MAPK/PI3K and is supportive for tumor growth [76]. Cixutumumab, a fully human IgG1 monoclonal antibody specifically targeting IGF-1R, has shown promising antiproliferative activity* in vitro* and clinically, in soft tissue sarcomas [77]. Cixutumumab was combined with paclitaxel in metastatic esophageal/GE junction cancer in a randomized phase II study [78]. The primary endpoint (PFS) was not met with median PFS reaching 2.6 months for paclitaxel alone and 2.3 months for paclitaxel + cixutumumab (90% CI 2–3.6 months, *P* = 0.72).

## 10. Plk-1 Targeted Therapy

Human polo-like kinase 1 (Plk-1) plays a pivotal role in mitosis in normal and malignant cells and preclinical studies have highlighted the importance of Plk-1 in tumor development [79, 80]. Plk-1 is overexpressed in gastric tumor tissue and is associated with worse prognosis [81]. Volasertib (BI 6727) is a potent Plk-1 inhibitor which induces cell cycle arrest and apoptosis and was administered in combination with an angiokinase inhibitor Nintedanib (BIBF 1120) in a phase I dose-escalation study. This found that it was well-tolerated and a single patient with gastric cancer achieved a partial response [82].

## 11. FGFR Therapy

Amplification of the FGFR2 (fibroblast growth factor receptor 2) has been associated with tumorigenesis and results in downstream activation of the MAPK/PI3K pathways [83]. The overexpression of members of the FGF family has been associated with tumor progression/metastasis and is associated with poor survival in gastric cancer [84]. There is currently a phase II trial investigating the effects of dovitinib, a dual VEGF, and FGFR inhibitor, in combination with docetaxel as second-line chemotherapy in gastric cancer [85].

## 12. PI3K/mTOR Therapy

The PI3K (phosphatidylinositol 3-kinase) pathway usually becomes activated through growth factor stimulation by EGFR, PDGFR, IGFR, or c-Met [86]. Once PI3K is activated, Akt becomes phosphorylated affecting cell cycle progression and angiogenesis and has antiapoptotic effects [87]. The mTOR pathway is a central kinase pathway that increases the production of proteins which regulate key cellular processes including metabolism, angiogenesis, and cell growth [88]. Phosphorylated mTOR, indicating constitutive activation, has been associated with tumor progression and worse prognosis in gastric cancer patients [89]. Genetic alterations affecting the PI3K/Akt/mTOR pathway are frequently found in gastric cancer [90–92]. There are several potential approaches to inhibit aberrant PI3K/Akt/mTOR signaling including specifically targeting a single component of the pathway, for example, mTOR inhibitors, or dual inhibitors, for example, dual PI3K/mTOR inhibitors. A phase Ib trial has been completed combining a PI3K inhibitor BYL719 with a HSP inhibitor AUY922 in metastatic gastric cancer patients whose tumors are HER2 amplified or who harbor a PI3K mutation [93]. Everolimus, an oral mTOR inhibitor, was investigated in advanced gastric cancer in the GRANITE-1 study [94]. Patients who had progressed after 1-2 lines of chemotherapy were randomized to receive everolimus or best supportive care (BSC). Median overall survival was 5.4 months with everolimus and 4.3 months with BSC (HR = 0.90, 95% CI 0.75–1.08, *P* = 0.124). There are also several ongoing trials comparing everolimus with chemotherapy (paclitaxel, capecitabine, and oxaliplatin) [95, 96].

## 13. Clinical Trial Design in Gastroesophageal Cancer

Many of the clinical trials involving molecularly targeted agents in gastroesophageal cancer have had initially promising results which do not persist later in larger phase II/III studies. Numbers of participants in these trials are small relative to other tumor types (e.g., breast or colon cancer) and this may explain, in part, the negative results. However, for the large part, these trials have been conducted in an unselected population. Designing clinical trials based on molecularly selected populations whose tumors overexpress a particular molecular marker, makes it more challenging to accrue, but represents a more meaningful approach. The PANGEA study (personalized antibodies for gastroesophageal adenocarcinoma) is an example of a trial that will prospectively determine whether assigning patients to targeted therapies based on the molecular profiling of their tumors, will impact median overall survival [97]. This innovative study design may represent the future of targeted therapy evaluation in gastroesophageal cancer where a variety of targeted therapies are now available to patients based on the individual molecular biology of their tumor.

## 14. Conclusion

The development of new therapies for advanced gastroesophageal cancer has been slow compared to other common tumors. Upper GI tumors represent diverse and heterogeneous diseases with multiple etiological factors including viral and bacterial infection via EBV and* Helicobacter pylori*, inherited familial syndromes, and other risk factors including diet, smoking, and alcohol consumption. Taken together, upper GI tumors are second only to lung cancer in terms of cancer related morbidity. In recent years incremental breakthroughs in our understanding of the molecular biology underlying the development of these cancers have taken place and these findings are now being translated into clinical research. High density genomic profiling arrays analyzing somatic copy-number alterations in gastroesophageal cancers have identified amplified genes in 37% of tumors, most notably,* ERBB2*,* FGFR1*,* FGFR2*,* EGFR*, and* MET* [27]. Until recently however the “one-size fits all approach” to enrolling patients on clinical trials has failed in gastroesophageal cancer as it has in other tumor types.

The Cancer Genome Atlas Research Network has recently identified other viable targets and it appears likely that the four molecular subtypes of gastric cancer described in this study should advance clinical research in the years to come. In addition to targeted agents there has been a rapid expansion in our understanding of the immune environment changes that occur in these tumors allowing them to avoid immune-editing. Future studies may combine targeted therapeutics with immunotherapies such as checkpoint inhibitors. Patient selection should be performed in all clinical trials to ensure that molecularly targeted therapies can fulfill their promise in upper GI tumors.

## Figures and Tables

**Table 1:** Current and recently completed phase III trials in gastric and gastroesophageal junction cancer.

Agent	Clinical trial	Randomization	*n*	NCT identifier
HER2 inhibitors				
Pertuzumab	JACOB	Pertuzumab in combination with Herceptin and chemotherapy	780	NCT01774786
T-DM1	GATSBY	T-DM1 with or without taxane.	412	NCT01641939
Trastuzumab	HELOISE	XP-T (standard versus high-dose)	400	NCT01450696

MET pathway inhibitors				
Rilotumumab	RILOMET-2	XP with or without rilotumumab	450	NCT02137343
Rilotumumab	RILOMET-1	ECX with or without rilotumumab	600	NCT01697072
Onartuzumab	METGASTRIC	FOLFOX with or without onartuzumab	800	NCT01662869

Cancer stemness inhibitor				
BBI608	BRIGHTER	Paclitaxel with or without BBI608	680	NCT02178956

EGFR inhibitors				
Panitumumab	REAL-3	EOX with or without panitumumab	574	NCT00824785
Cetuximab	EXPAND	XP with or without cetuximab	904	NCT00678535

Angiogenesis inhibitors				
Ramucirumab	REGARD	Ramucirumab versus BSC	355	NCT00917384
Ramucirumab	RAINBOW	Paclitaxel with or without ramucirumab	665	NCT01170663
Regorafenib	INTEGRATE	Regorafenib versus BSC	150	Actrn12612000239864

T-DM1: trastuzumab emtansine; XP: cisplatin, capecitabine; XP-T: cisplatin, capecitabine; T: trastuzumab; ECX: epirubicin, cisplatin, and capecitabine; FOLFOX: 5FU, folinic acid, and oxaliplatin; EOX: epirubicin, oxaliplatin, and capecitabine; BSC: best supportive care.
